# Fatal and non-fatal firearm-related injuries in Canada, 2016–2020: a population-based study using three administrative databases

**DOI:** 10.1186/s40621-023-00422-z

**Published:** 2023-02-14

**Authors:** Stephanie Toigo, Nathaniel J. Pollock, Li Liu, Gisèle Contreras, Steven R. McFaull, Wendy Thompson

**Affiliations:** Injury Surveillance, Public Health Agency of Canada, 785 Carling Avenue, Ottawa, ON K1S 5H4 Canada

**Keywords:** Firearms, Unintentional injury, Suicide, Self-harm, Homicide, Assault, Deaths, Hospitalizations, Emergency department visits

## Abstract

**Background:**

Firearms are a substantial cause of injury-related morbidity and mortality in Canada and globally, though evidence from contexts other than the USA is relatively limited. We examined deaths, hospitalizations and emergency department (ED) visits due to firearm-related injuries in Canada to identify population groups at increased risk of fatal and non-fatal outcomes.

**Methods:**

We conducted a population-based study using three national administrative databases on deaths, hospitalizations, and ED visits. ICD-10 codes were used to identify firearm-related injuries from January 1, 2016, through December 31, 2020. Fatal and non-fatal firearm injuries were classified as suicide/self-harm, homicide/assault, unintentional, undetermined or legal intervention injuries. We analyzed the data with counts, rates and proportions, stratified by sex, age group, province/territory, and year.

**Results:**

Over the 5-year period, we identified 4005 deaths, 3169 hospitalizations, and 2847 ED visits related to firearm injuries in various jurisdictions in Canada. Males comprised the majority of fatal and non-fatal injury cases. The highest rates of fatal and non-fatal firearm injuries were among 20- to 34-year-olds. The leading cause of fatal firearm injuries was self-harm (72.3%). For non-fatal firearm hospitalizations and ED visits, assault (48.8%) and unintentional injuries (62.8%) were the leading causes of injury. Rates varied by province and territory.

**Conclusions:**

Our results showed that males comprised the majority of fatal and non-fatal firearm injuries in Canada. The rates of both fatal and non-fatal firearm injuries were highest among the 20- to 34-year-old age group. This comprehensive overview of the epidemiology of firearm injuries in Canada provides baseline data for ongoing surveillance and policy evaluation related to public health interventions.

**Supplementary Information:**

The online version contains supplementary material available at 10.1186/s40621-023-00422-z.

## Background

Firearms are a highly lethal, yet modifiable, public health hazard (Mann and Michel 2016; McLeod et al. 2021), and are a leading cause of injury morbidity and mortality (Global Burden of Disease 2016 Injury Collaborators et al. 2016). In 2016, there were an estimated 251,000 firearm-related deaths worldwide, 64% of which were due to homicide, 27% to suicide, and 9% to unintentional (accidental) injuries (Global Burden of Disease 2016 Injury Collaborators et al. 2016). Although violence is the leading cause of firearm-related injuries and deaths (Global Burden of Disease 2016 Injury Collaborators et al. 2016), the majority occur outside of armed conflicts (Geneva Declaration 2022). According to global estimates, between 1990 and 2016, firearm mortality rates decreased from 4.2 to 3.4 deaths per 100,000 population; regional and national rates varied substantially by cause, likely related to geographic differences in firearm laws and sociodemographic risks (Global Burden of Disease 2016 Injury Collaborators et al. 2016). During this period, the population group most severely impacted by firearm mortality was males aged 20 to 24 years (Global Burden of Disease 2016 Injury Collaborators et al. 2016; Wintemute 2015).

Firearm mortality is disproportionately concentrated in low- and middle-income countries and in the USA (Global Burden of Disease 2016 Injury Collaborators et al. 2016). Of the 17 countries with firearm mortality rates above the 2016 global median, the majority were in Latin and South America, with high rates driven by homicides (Global Burden of Disease 2016 Injury Collaborators et al. 2016). The USA and Greenland were the only high-income nations with firearm mortality rates above the global median—they had the highest firearm suicide rates worldwide (Global Burden of Disease 2016 Injury Collaborators et al. 2016). The lowest rates of firearm mortality were in Asian countries, including China, Japan, and Singapore. In the USA, the most frequent causes of non-fatal firearm injury were assaults and unintentional injuries; suicide was the most frequent cause of fatal firearm injuries (Grinshteyn and Hemenway 2016; Gani et al. 2017; Avraham et al. 2018).

Compared with global medians, Canada had a significantly higher rate of suicide by firearm, but a significantly lower rate of homicide by firearm (Global Burden of Disease 2016 Injury Collaborators et al. 2016). Between 2010 and 2020, suicide was the leading cause of firearm death in Canada, with more than twice the number of deaths than homicide (6327 suicide deaths vs. 2258 homicide deaths) (Statistics Canada 2022a; Department of Justice 1999), though rates of firearm-related suicide and homicide decreased significantly between 1981 and 2018 (Langmann 2020; Liu et al. 2021). A recent study from the largest Canadian province, Ontario, identified 6,483 firearm-related injuries from 2002 through 2016; 33% of these injuries were due to assault; 31%, to self-harm; and 31%, to unintentional injuries (Gomez et al. 2020). Nearly half (42.3%) of these injuries were fatal, and case fatality was highest for self-harm injuries (91.7%) (Gomez et al. 2020).

The prevention of firearm injuries and deaths in Canada has been approached with a range of interventions, from local initiatives such as community firearm storage programs (Cormier 1998) and the distribution of free trigger locks (Nunatsiaq News 2019), to federal legislation and regulation on the sale, purchase, use, and licensing of firearms (Bennett et al. 2022; Ferguson and Koziarski 2019). A recent systematic review reported that firearm legislation in Canada has had an inconclusive effect on homicide rates, but has contributed to decreased suicide rates, albeit with a possible method substitution effect observed in several studies (Bennett et al. 2022). The impact of legislation and other interventions on non-fatal firearm injuries is unclear (Bennett et al. 2022).

Although firearms are a public health risk factor in Canada, the epidemiology of firearm injuries at the national level has not been thoroughly examined. Given this, there is a need to strengthen the knowledge base on firearm injuries and deaths in Canada, which in part can be achieved through an analysis of administrative health data. The objective of this study was to examine rates of firearm-related deaths and injury-related hospitalizations and emergency department (ED) visits in Canada, by cause (suicide/self-harm, homicide/assault, unintentional, undetermined and legal intervention), sex, age group, province/territory, and year.

## Methods

### Data sources and case definition

We analyzed administrative data on firearm deaths and firearm-related injury hospitalizations and ED visits for the 5-year period from January 1, 2016, through December 31, 2020. Deaths due to firearm injuries were from the Canadian Vital Statistics Death Database (CVSD). Hospital discharges related to firearm injuries were extracted from the Discharge Abstract Database (DAD). ED visits related to firearm injuries were extracted from the National Ambulatory Care Reporting System (NACRS). Statistics Canada is the data custodian for the CVSD and the Canadian Institute for Health Information (CIHI) is the data custodian for DAD and NACRS.

CVSD contains demographic and medical information, including cause, for all deaths in Canada. The vital statistics registry in each province and territory provides data from death registrations to Statistics Canada. Data for 2016 to 2020 were preliminary, as a result of recent improvements in methodology which shortened the duration of data collection compared to previous years. Due to the more timely nature of the data used in this study, some deaths may not have been captured or accurately classified at the time of release. Determination of cause of death is subject to change following medico-legal investigations, which can take up to 2 years (Statistics Canada 2021a, 2022a, 2022b). Due to limitations in the data-sharing agreements between the Public Health Agency of Canada and Statistics Canada, we were unable to access record-level data for some jurisdictions and therefore used publicly available aggregated CVSD data (Statistics Canada 2022a, 2022c). The publicly available data included death data for all provinces and territories in Canada from 2016 to 2020 except for Yukon, where only data from 2016 were available. We removed 2016 data for Yukon at the province/territory level, however, were unable to remove the same data from other breakdowns. Even without Yukon data, CVSD data used in this study had complete coverage for 99.9% of the Canadian population.

DAD collects administrative, demographic, and clinical information on hospital *separations* (also called *discharges*) from all provinces and territories, except Quebec. We analyzed DAD data from acute care inpatient and psychiatric hospital discharge records that covered 76.4% of the Canadian population.

NACRS contains administrative, demographic, and clinical data for emergency department visits and other forms of ambulatory care. The ED visit data in this study is from Ontario, Alberta and Yukon—the only jurisdictions that have mandated reporting, and therefore, complete population coverage for the study period. This data covers 49.4% of the Canadian population. Data sources and population coverage by province/territory are reported in Additional file 1.

Since the three data sources were not linked at the record level, we excluded select cases to avoid repeat-counting of a single injury event. For hospitalization data (DAD), we excluded patients who died during a hospital admission as we reasonably assumed they would be captured in the mortality data (CVSD). For ED visits (NACRS), we excluded patients who were hospitalized following an ED visit and those who died during the ED visit or during pre-hospital transport, as they would be captured in DAD and CVSD.

The International Classification of Diseases 10th revision (ICD-10) was used to classify the cause of death (fatal injuries) and morbidity that led to a hospitalization or an ED visit (non-fatal injuries). Causes of death or injury ICD codes were grouped by intent: suicide/self-harm (X72, X73, X74), homicide/assault (X93, X94, X95); unintentional injuries (W32, W33, W43); injuries with undetermined intent (Y22, Y23, Y24); and injuries related to legal interventions (Y35.0). Hospitalizations and ED visits involving injuries from BB guns, air guns or flare guns were excluded; however, they were not excluded from death data as ICD codes were not detailed enough to specify these non-powdered firearms. Previous research suggests that the majority of firearm injuries from non-powdered firearms occur in youth and young adults under the age of 25 and that the fatality rate from these injuries is very low (Freeman et al. 2017; Saunders et al. 2021; Cox et al. 2019).

All diagnostic fields were searched (25 in DAD and 10 in NACRS) to identify firearm-related injury cases, based on the selected ICD-10 codes. To avoid misclassification, patients with multiple firearm-related injury codes recorded in a single episode of care were excluded as this would preclude us from correctly identifying the cause of firearm injury. This led to seven hospitalizations and four ED visits being removed from our analysis.

### Statistical analysis

Fatal and non-fatal firearm injuries were examined as counts, proportions, and rates. Results were stratified by age, sex, cause, province/territory, and year. Age was stratified into five groups: 0 to 9 years, 10 to 19, 20 to 34, 35 to 64, and 65 or older; sex was defined as male/female. Based on Statistics Canada population estimates, age- and sex-specific rates per 100,000 population with 95% confidence intervals were calculated for firearm-related deaths, hospitalizations, and ED visits for a pooled 5-year period, 2016 to 2020. Age-standardized rates per 100,000 were directly standardized to the 2011 Canadian census population, with 95% confidence intervals.

The analyses were stratified by outcome (firearm-related mortality, hospitalization, and ED visit), cause, sex, age group, province/territory, and year. Hospitalization and ED data with small counts (one to four) were presented as ranges to reduce potential identification of individuals and comply with data-sharing agreements with CIHI and with the *Statistics Act of Canada *(Government of Canada 2017). Mortality data with small counts were not suppressed, as these data are publicly available (Statistics Canada 2022a). All analyses were conducted in SAS Enterprise Guide version 7.1 (SAS Institute Inc., Cary, NC, USA).

## Results

### Fatal and non-fatal firearm injuries by sex and age group

We identified 4005 deaths, 3169 hospitalizations, and 2847 ED visits related to firearm injuries in Canada from 2016 through 2020. Table 1 shows overall and sex-specific rates for firearm-related deaths, hospitalizations, and ED visits, by cause. For both fatal and non-fatal injuries, males comprised the majority of events (93.2% of fatalities, 90.3% of hospitalizations, and 87.6% of ED visits). Among males, there were 3732 firearm-related fatalities (4.1 per 100,000 population), 2860 hospitalizations (4.0 per 100,000), and 2494 ED visits (5.4 per 100,000). Among females, there were 273 fatalities (0.3/100,000 population), 309 hospitalizations (0.4 per 100,000), and 353 ED visits (0.7 per 100,000).

**Table 1:** Crude rates of fatal and non-fatal firearm injuries, by sex and cause, Canada 2016 to 2020

Cause of injury or death	Sex combined		Female		Male	
	*N* (%)	Rate per 100,000 population (95% CI)	*N* (%)	Rate per 100,000 population (95% CI)	*N* (%)	Rate per 100,000 population (95% CI)
Deaths*						
All causes	4005	2.17 (2.10, 2.24)	273 (6.82)	0.29 (0.26, 0.33)	3732 (93.18)	4.07 (3.94, 4.20)
Suicide	2895 (72.28)	1.57 (1.51, 1.62)	118 (43.22)	0.13 (0.10, 0.15)	2777 (74.41)	3.03 (2.92, 3.14)
Homicide	933 (23.30)	0.51 (0.47, 0.54)	144 (52.75)	0.15 (0.13, 0.18)	789 (21.14)	0.86 (0.80, 0.92)
Unintentional	74 (1.85)	0.04 (0.03, 0.05)	7 (2.56)	0.01 (0.00, 0.01)	67 (1.80)	0.07 (0.06, 0.09)
Undetermined	37 (0.92)	0.02 (0.01, 0.05)	0 (0.00)	0.00 (0.00, 0.00)	37 (0.99)	0.04 (0.03, 0.05)
Legal intervention	66 (1.65)	0.04 (0.03, 0.04)	4 (1.46)	0.00 (0.00, 0.01)	62 (1.66)	0.07 (0.05, 0.08)
*Hospitalizations*^*ƚ*^						
All causes	3169	2.22 (2.14, 2.30)	309 (9.75)	0.43 (0.38, 0.48)	2860 (90.25)	4.04 (3.89, 4.19)
Self-harm	206 (6.50)	0.14 (0.12, 0.16)	17 (5.50)	0.02 (0.01, 0.03)	189 (6.61)	0.27 (0.23, 0.31)
Assault	1546 (48.79)	1.08 (1.03, 1.14)	139 (44.98)	0.19 (0.16, 0.23)	1407 (49.20)	1.99 (1.88, 2.09)
Unintentional	1150 (36.29)	0.81 (0.76, 0.85)	129 (41.75)	0.18 (0.15, 0.21)	1021 (35.70)	1.44 (1.35, 1.53)
Undetermined	170 (5.36)	0.12 (0.10, 0.14)	17 (5.50)	0.02 (0.01, 0.03)	153 (5.35)	0.22 (0.18, 0.25)
Legal intervention	97 (3.06)	0.07 (0.05, 0.08)	7 (2.27)	0.01 (0.00, 0.02)	90 (3.15)	0.13 (0.10, 0.15)
*Emergency department visits*^*ǂ*^						
All causes	2847	3.06 (2.95, 3.18)	353 (12.40)	0.74 (0.66, 0.82)	2494 (87.60)	5.42 (5.20, 5.63)
Self-harm	56 (2.00)	0.06 (0.04, 0.08)	< 5 (< 5.0)	0.01 (0.00, 0.02)	50–54 (< 5.0)	0.11 (0.08, 0.14)
Assault	779 (27.36)	0.84 (0.78, 0.90)	83 (23.51)	0.18 (0.14, 0.22)	696 (27.91)	1.51 (1.40, 1.62)
Unintentional	1788 (62.80)	1.92 (1.84, 2.01)	238 (67.42)	0.51 (0.44, 0.57)	1550 (62.15)	3.37 (3.20, 3.53)
Undetermined	140 (4.92)	0.15 (0.13, 0.18)	20 (5.67)	0.04 (0.02, 0.06)	120 (4.81)	0.26 (0.21, 0.31)
Legal intervention	84 (2.95)	0.09 (0.07, 0.11)	5–9 (< 5.0)	0.02 (0.00, 0.03)	75–79 (< 5.0)	0.17 (0.13, 0.20)

Data sources: Deaths, Canadian Vital Statistics Death Database; Hospitalizations, Discharge Abstract Database; Emergency department visits, National Ambulatory Care Reporting System

*CI* Confidence interval

Sex-specific rates per 100,000 population are calculated using Statistics Canada population estimates from 2016 to 2020, with 95% CIs

*Including Yukon in 2016

^ƚ^Excluding Quebec from 2016 to 2020

^ǂ^Including only Alberta, Ontario and Yukon from 2016 to 2020

Hospitalization and emergency department data with small counts between one and four have been presented as a range

The leading cause of fatal firearm injuries was suicide (72.3%), followed by homicide (23.3%); relatively few deaths were attributable to unintentional injuries (1.9%), legal interventions (1.7%), or undetermined causes (0.9%). The most common causes of firearm hospitalizations were assaults (48.8%) and unintentional injuries (36.3%); relatively few were due to self-harm (6.5%), injuries of undetermined cause (5.4%), or injuries occurring in legal interventions (3.1%) (Table 1). In Alberta, Ontario, and Yukon combined, 62.8% of firearm-related ED visits were due to unintentional injuries; 27.4%, to assaults; 4.9%, to injuries of undetermined cause; 3.0%, to legal interventions; and 2.0%, to self-harm.

Table 2 shows age-specific rates for firearm deaths, hospitalizations, and ED visits, by cause. Almost half (44.4%) of firearm deaths were among 35- to 64-year-olds. However, 20- to 34-year-olds had significantly higher rates of homicide by firearm, compared with other age groups, and those aged 65 or older had significantly higher rates of suicide by firearm.

**Table 2:** Age-specific rates of fatal and non-fatal firearm injuries, by cause, Canada 2016 to 2020

Cause of injury or death	0–9 Years		10–19 Years		20–34 Years		35–64 Years		65 + Years	
	*N* (%)	Rate per 100,000 population (95% CI)	*N* (%)	Rate per 100,000 population (95% CI)	*N* (%)	Rate per 100,000 population (95% CI)	*N* (%)	Rate per 100,000 population (95% CI)	*N* (%)	Rate per 100,000 population (95% CI)
Deaths*										
All causes (*n* = 4005)	1 (0.02)	0.01 (0.00, 0.01)	242 (6.04)	1.18 (1.03, 1.33)	1102 (27.51)	2.93 (2.75, 3.10)	1778 (44.39)	2.37 (2.26, 2.48)	882 (22.02)	2.80 (2.61, 2.98)
Suicide	0	0.00	123 (4.25)	0.60 (0.50, 0.71)	529 (18.27)	1.41 (1.29, 1.53)	1429 (49.36)	1.90 (1.80, 2.00)	814 (28.12)	2.58 (2.40, 2.76)
Homicide	0	0.00	96 (10.29)	0.47 (0.38, 0.56)	500 (53.59)	1.33 (1.21, 1.44)	289 (30.98)	0.38 (0.34, 0.43)	48 (5.14)	0.15 (0.11, 0.20)
Unintentional	1 (1.35)	0.01 (0.00, 0.01)	15 (20.27)	0.07 (0.04, 0.11)	24 (32.43)	0.06 (0.04, 0.09)	21 (28.38)	0.03 (0.02, 0.04)	13 (17.57)	0.04 (0.02, 0.06)
Undetermined	0	0.0	6 (16.22)	0.03 (0.01, 0.05)	15 (40.54)	0.04 (0.02, 0.06)	11 (29.73)	0.01 (0.01, 0.02)	5 (13.51)	0.02 (0.00, 0.03)
Legal intervention	0	0.0	2 (3.03)	0.01 (0.00, 0.02)	34 (51.52)	0.09 (0.06, 0.12)	28 (42.42)	0.04 (0.02, 0.05)	2 (3.03)	0.01 (0.00, 0.02)
*Hospitalizations*^*ƚ*^										
All causes (*n* = 3169)	14 (0.44)	0.08 (0.04, 0.13)	422 (13.32)	2.61 (2.36, 2.85)	1698 (53.58)	5.70 (5.43, 5.97)	933 (29.44)	1.61 (1.51, 1.72)	102 (3.22)	0.41 (0.33, 0.50)
Self-harm	0 (0.00)	0.00	31 (15.05)	0.19 (0.12, 0.26)	50 (24.27)	0.17 (0.12, 0.21)	95 (46.12)	0.16 (0.13, 0.20)	30 (14.56)	0.13 (0.08, 0.17)
Assault	< 5 (< 5.0)	0.01 (0.00, 0.02)	202 (13.07)	1.25 (1.08, 1.42)	934 (60.41)	3.14 (2.94, 3.34)	392 (25.36)	0.68 (0.61, 0.75)	15–19 (< 5)	0.07 (0.04, 0.10)
Unintentional	10–14 (< 5.0)	0.08 (0.04, 0.13)	165 (14.35)	1.02 (0.86, 1.17)	580 (50.43)	1.95 (1.79, 2.11)	349 (30.35)	0.60 (0.54, 0.67)	40–44 (< 5)	0.18 (0.13, 0.24)
Undetermined	0 (0.00)	0.00	15–19 (10.0–14.0)	0.11 (0.06, 0.17)	81 (46.65)	0.27 (0.21, 0.33)	63 (37.06)	0.11 (0.08, 0.14)	5–9 (5.0–9.0)	0.03 (0.01, 0.06)
Legal intervention	0 (0.00)	0.00	5–9 (5.0–9.0)	0.04 (0.01, 0.07)	53 (54.64)	0.18 (0.13, 0.23)	34 (35.05)	0.06 (0.04, 0.08)	< 5 (< 5.0)	0.02 (0.00, 0.03)
*Emergency department visits*^*ǂ*^										
All causes (*n* = 2847)	36 (1.26)	0.35 (0.24, 0.47)	432 (15.17)	4.03 (3.65, 4.41)	1470 (51.63)	7.46 (7.08, 7.84)	813 (28.56)	2.17 (2.02, 2.32)	96 (3.37)	0.65 (0.52, 0.78)
Self-harm	0 (0.00)	0.00	< 5 (< 5.0)	0.04 (0.00, 0.07)	15 (26.79)	0.08 (0.04, 0.11)	27 (48.21)	0.07 (0.04, 0.10)	10–14 (15.0–19.0)	0.07 (0.03, 0.11)
Assault	< 5 (< 5.0)	0.01 (0.00, 0.03)	124 (15.92)	1.16 (0.95, 1.36)	460 (59.05)	2.33 (4.12, 2.55)	190 (24.39)	0.51 (0.43, 0.58)	< 5 (< 5.0)	0.03 (0.00, 0.05)
Unintentional	33 (1.85)	0.32 (0.21, 0.43)	280 (15.66)	2.61 (2.30, 2.92)	876 (48.99)	4.44 (4.15, 4.74)	521 (29.14)	1.39 (1.27, 1.51)	78 (4.36)	0.53 (0.41, 0.64)
Undetermined	< 5 (< 5.0)	0.02 (0.00, 0.05)	20 (14.29)	0.19 (0.10, 0.27)	71 (50.71)	0.36 (0.28, 0.44)	43 (30.71)	0.11 (0.08, 0.15)	< 5 (< 5.0)	0.03 (0.00, 0.05)
Legal intervention	0 (0.00)	0.00	< 5 (< 5.0)	0.04 (0.00, 0.07)	48 (57.14)	0.24 (0.17, 0.31)	30–34 (35.0–39.0)	0.09 (0.06, 0.11)	0 (0.00)	0.00

Data sources: Deaths, Canadian Vital Statistics Death Database; Hospitalizations, Discharge Abstract Database; Emergency department visits, National Ambulatory Care Reporting System

Age-specific rates per 100,000 population are calculated using Statistics Canada population estimates from 2016 to 2020, with 95% Cis

*CI* Confidence interval

*Including Yukon in 2016

^ƚ^Excluding Quebec from 2016 to 2020

^ǂ^Including only Alberta, Ontario and Yukon from 2016 to 2020

Hospitalization and emergency department data with small counts between one and four have been presented as a range

The 20 to 34 age group accounted for over half (53.6%) of firearm hospitalizations and had significantly higher rates of firearm-related assaults, unintentional injuries, undetermined injuries and legal intervention injuries, compared with other age groups (Table 2).

Patterns of firearm-related ED visits across age groups were similar to those of hospitalizations, with 20- to 34-year-olds accounting for more than half (51.6%) (Table 2). The 20 to 34 age group also had significantly higher rates of ED visits attributable to firearm-related assaults and to unintentional, undetermined, and legal intervention injuries.

### Fatal and non-fatal firearm injuries by province/territory and year

Table 3 shows crude rates per 100,000 population for firearm injury deaths, hospitalizations, and ED visits, by province and territory. Almost two-thirds (64.6%) of firearm deaths occurred in three provinces: Ontario, Quebec, and Alberta. Among the 10 provinces, Saskatchewan had the highest rate of firearm deaths (4.3 per 100,000); among the territories, Nunavut had the highest rate (22.7 per 100,000); both jurisdictions were higher than the national rate (2.1 per 100,000). The majority of firearm deaths in Saskatchewan (82.3%) and Nunavut (79.1%) were suicides. Manitoba and Nova Scotia (both 0.9 per 100,000) had the highest rates of firearm homicides, both above the national rate (0.5 per 100,000). Detailed results are available in Additional file 2.

**Table 3:** Crude rates of fatal and non-fatal firearm injuries, by province/territory, Canada 2016 to 2020

Provinces/territories	Deaths*^ƚ^		Hospitalizations^ǂ^		Emergency department visits^§^	
	*N* (%)	Rate per 100,000 population (95% CI)	*N* (%)	Rate per 100,000 population (95% CI)	*N* (%)	Rate per 100,000 population (95% CI)
Total	3935	2.13 (2.07, 2.20)	3169	2.22 (2.14, 2.30)	2847	3.06 (2.95, 3.18)
Newfoundland and Labrador	87 (2.21)	3.31 (2.61, 4.00)	26 (0.82)	0.99 (0.61, 1.37)	–	–
Prince Edward Island	9 (0.23)	1.18 (0.41, 1.95)	< 5 (< 5.0)	0.26 (0.00, 0.63)	–	–
Nova Scotia	163 (4.14)	3.41 (2.88, 3.93)	69 (2.18)	1.44 (1.10, 1.78)	–	–
New Brunswick	149 (3.79)	3.87 (3.25, 4.49)	53 (1.67)	1.38 (1.01, 1.75)	–	–
Quebec	711 (18.07)	1.70 (1.57, 1.82)	–	–	–	–
Ontario	1172 (29.78)	1.64 (1.55, 1.74)	1180 (37.24)	1.66 (1.56, 1.75)	1704 (59.85)	2.39 (2.28, 2.50)
Manitoba	227 (5.77)	3.37 (2.94, 3.81)	268 (8.46)	3.98 (3.51, 4.46)	–	–
Saskatchewan	248 (6.30)	4.29 (3.75, 4.82)	332 (10.48)	5.74 (5.12, 6.36)	–	–
Alberta	658 (16.72)	3.07 (2.83, 3.30)	739 (23.32)	3.44 (3.19, 3.68)	1127 (39.59)	5.25 (4.95, 5.60)
British Columbia	457 (11.61)	1.83 (1.67, 2.00)	472 (14.89)	1.89 (1.72, 2.06)	–	–
Yukon	–	–	10 (0.32)	4.99 (1.90, 8.08)	16 (0.56)	7.98 (4.07, 11.89)
Northwest Territories	11 (0.28)	4.89 (2.00, 7.78)	10 (0.32)	4.44 (1.69, 7.20)	–	–
Nunavut	43 (1.09)	22.7 (15.92, 29.49)	5–9 (< 5.0)	4.22 (1.30, 7.15)	–	–

Data sources: Deaths, Canadian Vital Statistics Death Database; Hospitalizations, Discharge Abstract Database; Emergency department visits, National Ambulatory Care Reporting System

Crude rates per 100,000 population are calculated using Statistics Canada population estimates from 2016 to 2020, with 95% CIs

*CI* Confidence interval

*Excluding Yukon from 2016 to 2020 (*n* = 4)

^ƚ^Counts, proportions, and rates do not include injuries occurring in a legal setting (*n* = 66)

^ǂ^Excluding Quebec from 2016 to 2020

^§^Including only Alberta, Ontario and Yukon from 2016 to 2020

–Data not available

Hospitalization and emergency department data with small counts between one and four have been presented as a range

The majority (75.5%) of firearm-related hospitalizations occurred in Ontario, Alberta, and British Columbia. Among the nine provinces analyzed, Saskatchewan had the highest rate of firearm injury hospitalizations (5.7 per 100,000) and was higher than the national rate (2.2 per 100,000). Among the territories, Yukon had the highest rate (5.0 per 100,000). In both Saskatchewan (43.4%) and Yukon (40.0%), unintentional injuries were the most prevalent cause of firearm-related hospitalizations.

Among firearm-related injury ED visits in Alberta, Ontario, and Yukon, the highest rate was in Yukon (8.0 per 100,000). Unintentional injuries were the leading reason for firearm-related ED visits: 68.8% in Alberta, 58.5% in Ontario, and 100.0% in Yukon. From 2016 to 2019, the number of firearm-related fatalities remained relatively stable. Firearm-related hospitalizations rose slightly during the period (Table 4).

**Table 4:** Age-standardized rates of fatal and non-fatal firearm injuries, by year and cause, Canada 2016 to 2020

Years	All causes*		Suicide/self-harm		Homicide/assault		Unintentional		Undetermined	
	*N* (%)	Rate per 100,000 population (95% CI)	*N* (%)	Rate per 100,000 population (95% CI)	*N* (%)	Rate per 100,000 population (95% CI)	*N* (%)	Rate per 100,000 population (95% CI)	*N* (%)	Rate per 100,000 population (95% CI)
*Deaths*^*ƚ*^										
2016	738 (18.43)	2.02 (1.88, 2.17)	570 (19.69)	1.56 (1.43, 1.69)	138 (14.79)	0.38 (0.31, 0.44)	15 (20.27)	0.04 (0.02, 0.06)	5 (13.51)	0.01 (0.00, 0.02)
2017	861 (21.50)	2.34 (2.18, 2.50)	616 (21.28)	1.67 (1.54, 1.80)	210 (22.51)	0.58 (0.50, 0.65)	12 (16.22)	0.03 (0.01, 0.05)	7 (18.92)	0.02 (0.01, 0.03)
2018	839 (20.95)	2.24 (2.09, 2.39)	598 (20.66)	1.59 (1.46, 1.72)	208 (22.29)	0.56 (0.48, 0.64)	8 (10.81)	0.02 (0.01, 0.03)	8 (21.62)	0.02 (0.01, 0.03)
2019	849 (21.20)	2.23 (2.08, 2.38)	602 (20.79)	1.57 (1.45, 1.70)	206 (22.08)	0.55 (0.47, 0.62)	20 (27.03)	0.05 (0.03, 0.07)	13 (35.14)	0.03 (0.01, 0.05)
2020^§^	718 (17.93)	1.85 (1.72, 1.99)	509 (17.58)	1.30 (1.19, 1.42)	171 (18.33)	0.45 (0.38, 0.52)	19 (25.68)	0.05 (0.03, 0.07)	4 (10.81)	0.01 (0.00, 0.02)
*Hospitalizations*^*ǂ*^										
2016	553 (17.45)	1.96 (1.80, 2.12)	39 (18.93)	0.14 (0.10, 0.19)	288 (18.64)	1.02 (0.90, 1.13)	188 (16.35)	0.67 (0.57, 0.77)	28 (16.47)	0.10 (0.06, 0.13)
2017	543 (17.13)	1.91 (1.75, 2.07)	41 (19.90)	0.14 (0.10, 0.19)	265 (17.15)	0.93 (0.82, 1.04)	191 (16.61)	0.68 (0.58, 0.77)	24 (14.12)	0.08 (0.05, 0.12)
2018	612 (19.31)	2.12 (1.95, 2.29)	38 (18.45)	0.14 (0.09, 0.18)	296 (19.16)	1.03 (0.91, 1.14)	221 (19.22)	0.77 (0.66, 0.87)	39 (22.94)	0.14 (0.09, 0.18)
2019	728 (22.97)	2.48 (2.30, 2.66)	39 (18.93)	0.13 (0.09, 0.18)	361 (23.30)	1.23 (1.10, 1.35)	258 (22.43)	0.89 (0.78, 0.99)	42 (24.71)	0.14 (0.10, 0,19)
2020^§^	733 (23.13)	2.46 (2.28, 2.64)	49 (23.79)	0.17 (0.12, 0.21)	336 (21.75)	1.13 (1.00, 1.24)	292 (25.39)	0.98 (0.87, 1.09)	37 (21.76)	0.13 (0.09, 0.17)

Data sources: Deaths, Canadian Vital Statistics Death Database; Hospitalizations, Discharge Abstract Database

Age-standardized rates per 100,000 population are standardized to the 2011 Canadian population using direct standardization, with 95% CIs

*CI* Confidence interval

*Including legal interventions

^ƚ^Including Yukon in 2016

^ǂ^Excluding Quebec from 2016 to 2020

^§^2020 data are provisional and subject to be updated due to report delay; therefore, results for 2020 are likely underestimated

## Discussion

From 2016 through 2020, there were 4005 deaths, 3169 hospitalizations, and 2847 ED visits related to firearm injuries in Canada. The most prevalent cause of firearm death was suicide; assault and unintentional injuries were the most frequent causes of firearm-related hospitalizations and ED visits. The majority of firearm injuries were among males. The prevalence of fatal firearm injuries was highest in the 35- to 64-year-old age group, whereas non-fatal firearm injuries were more prevalent in the 20- to 34-year-old age group.

Our study adds Canadian data to the existing evidence demonstrating that males have significantly higher rates of firearm-related injuries compared with females (Saunders et al. 2021; Kaufman et al. 2021; Johns Hopkins-Center for Gun Violence Solutions 2020). Firearm homicide rates peaked in the 20- to 34-year-old age group, whereas firearm suicide rates were highest at age 65 or older (Kaufman et al. 2021; Allareddy et al. 2012). The higher firearm suicide rates among older adults may, in part, be explained by previous research reporting that guns are the most prevalent suicide method used by elderly men (Kaufman et al. 2021; Johns Hopkins-Center for Gun Violence Solutions 2020; Lustenberger et al. 2011; Price and Khubchandani 2021). Evidence suggests that physical and mental decline among older adults may increase suicide risk (Price and Khubchandani 2021; Betz et al. 2018; Morgan et al. 2019). Elderly men may be especially vulnerable after the onset of diseases associated with chronic pain such as arthritis and cancer, neurodegenerative diseases that impair cognitive function such as dementia, and social risks related to isolation and grief (Price and Khubchandani 2021; Betz et al. 2018; Morgan et al. 2019). Those in rural areas may experience additional risks related to increased access to firearms, especially for hunting (Kaufman et al. 2021; Wexler et al. 2022; Pollock et al. 2020), though we did not examine rurality in this study.

Overall, our analysis showed relatively low rates of fatal and non-fatal firearm injuries among children, compared to other age groups. Youth also had relatively low rates of fatal firearm injuries, but higher rates of non-fatal firearm injuries. Other Canadian studies have yielded mixed results. Surveillance data showed that firearm injuries and deaths among youth declined from 1979 to 2003 (Pan et al. 2007). More recent studies have shown few firearm injuries among younger children, but a rising incidence among youth (Saunders et al. 2021; Cox et al. 2019). However, the majority of those injuries involved non-powder firearms such as BB guns and airguns (Saunders et al. 2021; Cox et al. 2019). By contrast, US studies reported substantially higher rates of fatal and non-fatal firearm injuries for all causes among children and youth (Allareddy et al. 2012; Fowler et al. 2017). The lower rates of firearm injuries among children and youth in Canada compared to the USA may be due to differences in the prevalence of household firearm ownership, access, and storage practices (Gabor et al. 2001; Frappier et al. 2005).

Across Canada, rates of firearm mortality and hospitalization varied. Some of this heterogeneity, especially for suicide/self-harm and unintentional injuries, may reflect rural–urban differences in firearm ownership. Access to a gun in the household is a leading risk factor for fatal and non-fatal firearm-related injuries (Gabor et al. 2001; Frappier et al. 2005; Canadian Paediatric Society 2022). Licensing data for 2019 show that the provinces and territories with the highest firearm mortality rates also had the highest prevalence of firearm licenses (Department of Justice 1999; Royal Canadian Mounted Police 2020). We did not directly examine this relationship as licensing data may not be a useful proxy for firearm access, and no recent survey data are available about ownership and storage practices in Canada. This evidence gap makes it challenging to assess the relationship between firearm access or ownership and gun-related injuries at the individual and population levels, as has been done in the USA (Mann and Michel 2016), and limits opportunities for evaluating the impact of firearm policy and legislation.

Our study revealed that Manitoba and Nova Scotia had the highest rates of firearm homicides from 2016 to 2020, which is consistent with reports based on police data (Allen 2022). During the study period, several mass shootings occurred in Canada. Three of the most severe incidents include the Nova Scotia mass shooting in 2020, where 23 people died and three had non-fatal injuries; the Quebec City mosque shooting in 2017, where six people died and five were injured, and the mass shooting in Toronto’s Danforth neighborhood in 2018, where two people died and 13 were injured (Saskatoon StarPhoenix 2022). Homicide rates across Canada are relatively low; therefore, mass causality events such as those that occurred during the period covered by our data substantially increase firearm homicides rates, especially in smaller provinces and territories; this may, in part, account for the relatively high rate in Nova Scotia (Allen 2022). The elevated homicide rate in Manitoba may be related to the overall higher rate of firearm-related crime (Allen 2022). Other factors that we were unable to investigate, such as rurality, income, and education, may also influence differences across provinces and territories (Kegler et al. 2021; Patel et al. 2021).

This study had several limitations related to administrative data. One issue is that mortality data are subject to classification bias. Underlying cause of death in vital statistics mortality data (CVSD) is subject to revision following investigation by a coroner or medical examiner (Statistics Canada 2021b). The investigation process can take up to 18 months before a final cause is assigned, and additional time is needed to update vital statistics registries. As a result, the mortality data in this study may have underestimated rates for some firearm-related causes, such as homicide and suicide, and overestimated others such as undetermined or unintentional deaths.

Another limitation concerns the accuracy of ICD-10 codes. Compared with clinical chart reviews, ICD codes have only moderate agreement on firearm injury intent (Donnelly et al. 2022). Consequently, both homicide/assault and suicide/self-harm may be misclassified as unintentional or undetermined injuries (or vice versa) when evidence about intent at the time of injury is lacking (Barber and Hemenway 2011). Previous research has also highlighted that the misclassification of non-fatal firearm injuries can lead to an undercount of cases (Magee et al. 2021; Post et al. 2019). Often patient self-report is the main source of information, as other sources such as police report or medico-legal investigations may not be available at the time of treatment to corroborate circumstances of the injury. Additionally, utilizing administrative databases to capture non-fatal injuries may lead to results that are biased toward more severe injuries as only injuries that required medical attention in a hospital setting are captured. Due to the nature of firearm-related injuries, there may also be individuals who forgo medical care for fear of legal consequences.

A related challenge is that administrative data on homicide/assault may not consistently or accurately capture secondary causes of death such as intimate partner violence (IPV) (Espinoza and Camacho 2005) or child maltreatment (Herman-Giddens et al. 1999). In the USA, 55% of homicide deaths among women were related to IPV (Petrosky et al. 2022), and firearms were the most common weapons used in IPV-related homicides (Velopulos et al. 2019). Such disaggregation was not possible with our data. However, distinguishing between specific types of violence-related injuries is important because it can inform prevention efforts by parsing out risks related to community and family violence, especially when gendered differences exist.

A limitation of all three datasets was the varied geographic coverage. The publicly available CVSD data we used did not contain mortality data for Yukon from 2017 to 2020, and the DAD hospitalization data did not include Quebec. NACRS had complete data coverage for only two provinces and one territory—Alberta, Ontario and Yukon. Nonetheless, the jurisdictions where injury and mortality data were available had a comprehensive capture and the results were robust and should be considered generalizable to the provinces and territories they include. The use of data from the pandemic period in 2020 (mid-March through December) may have impacted hospitalization and ED visit volumes toward the end of the study period (Canadian Institute for Health Information 2022).

## Conclusion

Firearm injuries are responsible for considerable morbidity and mortality in Canada. Over the 2016 to 2020 period, rates of fatal and non-fatal firearm injuries were highest among males and 20- to 34-year-olds. The most frequent causes of non-fatal firearm injuries were unintentional injuries and assaults, whereas the most frequent cause of fatal injuries was suicide. Much of the epidemiological literature on firearm injury focuses on the USA; therefore, providing Canadian evidence is valuable for a comparative perspective. Future research employing qualitative data may provide additional details about the circumstances and intent of firearm injuries and deaths, and about characteristics that put some population subgroups at increased risk. This analysis contributes to the firearm injury literature and to evidence-based efforts aimed at injury prevention and firearm regulation.

## Supplementary Information

**Additional file 1: Table S1.** Data sources and population coverage by province/territory. This table provides an overview of the three data sources used for this analysis and the population coverage of each, by province/territory.

**Additional file 2: Table S2.** Crude rates of fatal firearm injuries, by province/territory and cause, Canada, 2016 to 2020. This is an additional table that presents crude rates as well as prevalence of fatal firearm injuries in Canada across province/territory by cause of injury from 2016 to 2020. The data in this table are from the Canadian Vital Statistics Death Database and does not include data from Yukon during this period.

## Data Availability

Access to programming code and aggregated data can be made available upon request through the corresponding author.

## Abbreviations

ED: Emergency Department
CVSD: Canadian Vital Statistics Death Database
DAD: Discharge Abstract Database
NACRS: National Ambulatory Care Reporting System
CIHI: Canadian Institute for Health Information
ICD-10: International Classification of Diseases, 10^th^ revision
IPV: Intimate partner violence
