# Supplementary material for: Fatal and non-fatal firearm-related injuries in Canada, 2016–2020: a population-based study using three administrative databases
Source: Inj Epidemiol. 2023 Feb 14;10:10. doi: 10.1186/s40621-023-00422-z (PMC9930327; doi:10.1186/s40621-023-00422-z)
Supplement: Supplementary file 1 — Additional file 1: Table S1. Data sources and population coverage by province/territory. This table provides an overview of the three data sources used for this analysis and the population coverage of each, by province/territory. [file 40621_2023_422_MOESM1_ESM.docx]

**Additional file 1:**

| **Table S1.** Data sources and population coverage by province/territory, Canada 2016 to 2020 | | | | | | | | | | | | | | | |
| --- | --- | --- | --- | --- | --- | --- | --- | --- | --- | --- | --- | --- | --- | --- | --- |
| **Data source** | **Canadian population coverage (%)** | **NL** | **PE** | **NS** | **MB** | **NB** | **QC** | **ON** | **MB** | **SK** | **AB** | **YT** | **NT** | **NU** |  |
| Deaths (CVSD) | 99.9% | ✓ | ✓ | ✓ | ✓ | ✓ | ✓ | ✓ | ✓ | ✓ | ✓ | ✓^*^ | ✓ | ✓ |  |
| Hospitalizations (DAD) | 76.4% | ✓ | ✓ | ✓ | ✓ | ✓ |  | ✓ | ✓ | ✓ | ✓ | ✓ | ✓ | ✓ |  |
| Emergency department visits (NACRS) | 49.4% |  |  |  |  |  |  | ✓ |  |  | ✓ | ✓ |  |  |  |
| **Data sources:** Deaths, Canadian Vital Statistics Death Database; Hospitalizations, Discharge Abstract Database; Emergency Department Visits, National Ambulatory Care Reporting System  *Publicly available CVSD data for Yukon (YT) is available in 2016, but not from 2017 to 2020. | | | | | | | | | | | | | | | |
