# Supplementary material for: Fatal and non-fatal firearm-related injuries in Canada, 2016–2020: a population-based study using three administrative databases
Source: Inj Epidemiol. 2023 Feb 14;10:10. doi: 10.1186/s40621-023-00422-z (PMC9930327; doi:10.1186/s40621-023-00422-z)
Supplement: Supplementary file 2 — Additional file 2: Table S2. Crude rates of fatal firearm injuries, by province/territory and cause, Canada, 2016 to 2020. This is an additional table that presents crude rates as well as prevalence of fatal firearm injuries in Canada across province/territory by cause of injury from 2016 to 2020. The data in this table are from the Canadian Vital Statistics Death Database and does not include data from Yukon during this period. [file 40621_2023_422_MOESM2_ESM.docx]

**Additional file 2:**

| **Table S2.** Crude rates of fatal firearm injuries, by province/ territory and cause, Canada, 2016 to 2020 | | | | | | | | |
| --- | --- | --- | --- | --- | --- | --- | --- | --- |
|  | **Suicide** | | **Homicide** | | **Unintentional** | | **Undetermined** | |
| **Provinces/ Territories** | **N (%)** | **Rate per 100,000 population**  **(95% CI)** | **N (%)** | **Rate per 100,000 population**  **(95% CI)** | **N (%)** | **Rate per 100,000 population**  **(95% CI)** | **N (%)** | **Rate per 100,000 population**  **(95% CI)** |
| **Total** | **2891** | **1.57 (1.51, 1.62)** | **933** | **0.51 (0.47, 0.54)** | **74** | **0.04 (0.03, 0.05)** | **37** | **0.02 (0.01, 0.03)** |
| British Columbia | 383 (13.25) | 1.54 (1.38, 1.69) | 60 (6.43) | 0.24 (0.18, 0.30) | 11 (14.86) | 0.04 (0.02, 0.07) | 3 (8.11) | 0.01 (0.00, 0.03) |
| Alberta | 514 (17.78) | 2.40 (2.19, 2.60) | 139 (14.90) | 0.65 (0.54, 0.76) | 1 (1.35) | 0.00 (0.00, 0.01) | 4 (10.81) | 0.02 (0.00, 0.04) |
| Saskatchewan | 204 (7.06) | 3.53 (3.04, 4.01) | 35 (3.75) | 0.61 (0.40, 0.81) | 8 (10.81) | 0.14 (0.04, 0.23) | 1 (2.70) | 0.02 (0.00, 0.05) |
| Manitoba | 151 (5.22) | 2.24 (1.89, 2.60) | 62 (6.65) | 0.92 (0.69, 1.15) | 9 (12.16) | 0.13 (0.05, 0.22) | 5 (13.51) | 0.07 (0.01, 0.14) |
| Ontario | 680 (23.52) | 0.95 (0.88, 1.03) | 469 (50.27) | 0.66 (0.60, 0.72) | 12 (16.22) | 0.02 (0.01, 0.03) | 11 (29.73) | 0.02 (0.01, 0.02) |
| Quebec | 587 (20.30) | 1.40 (1.29, 1.51) | 96 (10.29) | 0.23 (0.18, 0.28) | 20 (27.03) | 0.05 (0.03, 0.07) | 8 (21.62) | 0.02 (0.01, 0.03) |
| New Brunswick | 121 (4.19) | 3.14 (2.58, 3.70) | 25 (2.68) | 0.65 (0.39, 0.90) | 3 (4.05) | 0.08 (0.00, 0.17) | 0 (0.00) | 0.00 |
| Nova Scotia | 118 (4.08) | 2.47 (2.02, 2.91) | 42 (4.50) | 0.88 (0.61, 1.14) | 2 (2.70) | 0.04 (0.00, 0.10) | 1 (2.70) | 0.02 (0.00, 0.06) |
| Newfoundland and Labrador | 80 (2.77) | 3.04 (2.37, 3.71) | 3 (0.32) | 0.11 (0.00, 0.24) | 3 (4.50) | 0.11 (0.00, 0.24) | 1 (2.70) | 0.04 (0.00, 0.11) |
| Prince Edward Island | 8 (0.28) | 1.05 (0.32, 1.78) | 1 (0.11) | 0.13 (0.00, 0.39) | 0 (0.00) | 0.00 | 0 (0.00) | 0.00 |
| Yukon | -- | -- | -- | -- | -- | -- | -- | -- |
| Northwest Territories | 11 (0.38) | 4.89 (2.00, 7.78) | 0 (0.00) | 0.00 | 0 (0.00) | 0.00 | 0 (0.00) | 0.00 |
| Nunavut | 34 (1.18) | 17.95 (11.92, 23.99) | 1 (0.11) | 0.53 (0.00, 1.56) | 5 (6.76) | 2.64 (0.33, 4.95) | 3 (8.11) | 1.58 (0.00, 3.38) |
| **Data sources:** Deaths, Canadian Vital Statistics Death Database  **Abbreviations:** CI, Confidence interval  **Notes:** Crude rates per 100,000 population are calculated using Statistics Canada population estimates from 2016 to 2020, with 95% CIs.  ^*^Excluding Yukon from 2016 to 2020  -- Data not available | | | | | | | | |
